# Laboratory-based surveillance of antimicrobial non-susceptibility in *Enterobacter* species over 12 years at a tertiary-care hospital in Saudi Arabia

**DOI:** 10.1371/journal.pone.0358963

**Published:** 2026-10-16

**Authors:** Yahya Shabi, Fatimah Mohmmed Ali Asiri, Khalifa Binkhamis, Ali M. Somily, Mona M. Alshahrani, Tarik Alazraqi, Safia Abdullah Mohammed, Sara Habbash, Ali Al Bshabshe, Soliuman Mohamed, Abdullah Algarni, Ahmadiyah Siddiq, Abdulah O. S. Bawazeer, Abdulah J. Alqahtani, Abdullah A. Al-Shahrani

**Affiliations:** 1 Department of Microbiology and Clinical Parasitology, College of Medicine, King Khalid University, Abha, Saudi Arabia; 2 Khamis Mushait General Hospital, Khamis Mushait, Saudi Arabia; 3 Department of Pathology, College of Medicine, King Saud University, Riyadh, Saudi Arabia; 4 Consultant Nephrology and Assistant Professor at King Khalid University, Abha, Saudi Arabia; 5 Department of Internal Medicine, Aseer Central Hospital, Abha, Saudi Arabia; 6 Department of Medicine, College of Medicine, King Khalid University, Abha, Saudi Arabia; 7 Department of Haematology and Immunohaematology, Faculty of Medical Laboratory Sciences, University of Khartoum, Khartoum, Sudan; 8 Department of Family Medicine, Aseer Central Hospital, Abha, Saudi Arabia; 9 Department of Internal Medicine, College of Medicine, King Khalid University, Abha, Saudi Arabia; 10 Department of Microbiology, Aseer Central Hospital, Abha, Saudi Arabia; Universidad San Francisco de Quito, ECUADOR

## Abstract

**Background:**

*Enterobacter* species are increasingly associated with antimicrobial non-susceptibility, but long-term surveillance of temporal trends across antibiotic classes is limited in southern Saudi Arabia.

**Methods:**

We retrospectively studied non-duplicate *Enterobacter* isolates from adults at a tertiary-care hospital in southern Saudi Arabia (August 2012–August 2024), interpreted by contemporaneous CLSI criteria. Temporal trends were modelled by logistic regression as the annual percent change (APC) in the odds of non-susceptibility, adjusted for care setting, specimen source, age, and sex. Carbapenem-resistant Enterobacterales (CRE) and difficult-to-treat resistance (DTR; Kadri criteria) were analysed over time; sensitivity analyses excluded partial years and used breakpoint-comparable periods.

**Results:**

Of 1,319 *Enterobacter* isolates, 91.8% belonged to the *Enterobacter cloacae* complex. Adjusted trends declined for amikacin (APC −6.1%), gentamicin (−9.4%) and cefepime (−9.1%) and rose for carbapenems (+13.1% to +15.8%), levofloxacin (+6.3%) and piperacillin–tazobactam (+7.0%), with ciprofloxacin stable. The levofloxacin and piperacillin–tazobactam increases did not persist in breakpoint-comparable analyses and are not unequivocal biological increases. Inpatient isolates were more non-susceptible than outpatient isolates by approximately 16–39 percentage points (gentamicin 31.7% vs 14.8%; piperacillin–tazobactam 38.7% vs 23.1%; cefotaxime 63.9% vs 41.5%; cefepime 45.7% vs 28.8%; ceftazidime 61.4% vs 35.3%; ceftriaxone 57.4% vs 18.8%; all p ≤ 0.003). CRE rose early and remained common (APC + 22.8%; 28.7% overall), whereas DTR rose later and stayed infrequent (7.5% of eligible isolates; APC + 16.4%; highest annual value 20.2% in 2023); this difference in timing and overall frequency, not the partially sampled year 2024, indicates dissociation between carbapenem non-susceptibility and loss of all first-line agents.

**Conclusion:**

Non-susceptibility among *Enterobacter* species was heterogeneous and antibiotic-specific. The dissociation between CRE and DTR in timing and overall frequency supports using clinically meaningful phenotypes alongside antibiotic-specific trend analysis for stewardship and reporting; the levofloxacin and piperacillin–tazobactam increases warrant caution, as they did not persist in breakpoint-comparable analyses.

## Introduction

Species of the genus *Enterobacter* are widely distributed in environmental reservoirs and may form part of the human gastrointestinal microbiota, but they have increasingly emerged as clinically significant opportunistic pathogens in community-onset and healthcare-associated infections, particularly among hospitalized and immune-compromised patients. They are implicated in urinary tract infections, bloodstream infections, pneumonia, intra-abdominal infections, device-associated infections, and nosocomial outbreaks in high-risk healthcare settings [1]. Within this genus, the *Enterobacter cloacae* complex is a genetically heterogeneous group that accounts for a substantial proportion of clinically relevant *Enterobacter* infections and is particularly prominent in healthcare-associated outbreaks, including neonatal intensive care unit outbreaks [1–3].

Taxonomic refinement has demonstrated that the *Enterobacter cloacae* complex comprises multiple closely related species with distinct genomic characteristics, including *Enterobacter cloacae*, *Enterobacter hormaechei*, *Enterobacter asburiae*, *Enterobacter kobei*, *Enterobacter ludwigii*, *Enterobacter roggenkampii*, and related taxa [2,4,5]. Whole-genome sequencing and genome-based classification have improved species-level resolution within the complex and have shown clinically relevant differences in antimicrobial non-susceptibility patterns and virulence-associated traits that are not reliably captured by conventional phenotypic identification methods [5,6].

Clinically relevant *Enterobacter* species exhibit intrinsic non-susceptibility to aminopenicillins and early-generation cephalosporins, primarily mediated by chromosomally encoded AmpC beta-lactamases. Inducible, derepressed, or hyperproduced AmpC can limit the reliability of third-generation cephalosporins and beta-lactam/beta-lactamase inhibitor combinations, complicating empirical and definitive treatment strategies [1,2,7]. In addition, *Enterobacter* species may acquire plasmid-mediated extended-spectrum beta-lactamases, carbapenemases, porin alterations, efflux-mediated mechanisms, and quinolone resistance determinants, producing multidrug non-susceptibility or difficult-to-treat resistance (DTR) phenotypes that restrict first-line therapeutic options [8–11].

Escalating antimicrobial non-susceptibility has increased the clinical burden posed by opportunistic Enterobacterales, including the *Enterobacter* species. In particular, carbapenem-resistant Enterobacterales (CRE) are recognized as a critical public health threat, with well-documented associations with increased mortality, prolonged hospitalization, and severely limited therapeutic options [12,13]. Surveillance data from international and regional programs have documented increasing antimicrobial non-susceptibility among Enterobacterales, with substantial geographic variability across regions and health system contexts [13,14]. Standardized interpretive criteria and susceptibility testing frameworks, such as those provided by CLSI and EUCAST, are essential for accurate detection, reporting, and comparison of non-susceptibility trends across institutions and regions [15,16].

Recent studies from Saudi Arabia have highlighted the clinical and epidemiological importance of the *Enterobacter* species in healthcare settings, driven by the detection of multidrug non-susceptibility and extensively drug non-susceptibility isolates and the emergence of high-risk non-susceptibility determinants, particularly among members of the *Enterobacter cloacae* complex [17–19]. Molecular epidemiological studies from tertiary care hospitals in Saudi Arabia have identified carbapenemase-producing *Enterobacter* species, including isolates harboring *bla*NDM and other clinically significant non-susceptibility genes, underscoring their clinical relevance and the role of mobile non-susceptibility determinants in regional healthcare settings [17,19,20]. Clinical outcome studies indicate that infections caused by non-susceptible *Enterobacter* species are associated with adverse outcomes and complex therapeutic challenges. This burden is particularly pronounced among carbapenem non-susceptible isolates and among infections caused by members of the *Enterobacter cloacae* complex, where uncertainty persists regarding optimal carbapenem-sparing treatment strategies [12,18,21].

Beyond antimicrobial non-susceptibility, members of the genus *Enterobacter* can form biofilms on abiotic surfaces and medical devices, a trait that may contribute to environmental persistence in healthcare settings and has been most extensively characterized among members of the *Enterobacter cloacae* complex [22]. These factors underscore the importance of robust local epidemiological surveillance to support antimicrobial stewardship and infection prevention efforts while providing context for evidence-based clinical practice [13,14].

Despite a growing global and regional literature, long-term, institution-level analyses examining temporal trends in *Enterobacter* species distribution, antimicrobial susceptibility patterns, and clinically relevant non-susceptibility phenotypes remain limited, particularly in southern Saudi Arabia. Decade-scale data from tertiary-care settings serving geographically distinct regions of the country are needed to contextualize local epidemiology, guide laboratory reporting, and support antimicrobial stewardship in alignment with international standards. The present study therefore aimed to conduct long-term laboratory-based surveillance of temporal trends in phenotypic antimicrobial non-susceptibility across routinely tested antibiotics among *Enterobacter* species and to describe the longitudinal behavior of clinically relevant non-susceptibility phenotypes, including CRE and DTR, in a tertiary-care hospital in southern Saudi Arabia.

## Materials and methods

### Study design and setting

This retrospective longitudinal surveillance study, based on serial cross-sectional analyses, was conducted at Aseer Central Hospital, a tertiary care and regional referral center in Abha, southern Saudi Arabia. The hospital provides diagnostic and referral services for the entire Aseer region. The study included all *Enterobacter* isolates recovered between August 2012 and August 2024 from clinical specimens processed in the Department of Microbiology.

This study was designed as a laboratory-based surveillance analysis and did not aim to assess clinical outcomes, treatment efficacy, transmission dynamics, or molecular mechanisms of antimicrobial non-susceptibility.

### Data collection and isolate selection

Microbiological data were retrieved from the Laboratory Information System (LIS). All *Enterobacter* isolates obtained from clinical specimens during the study period were screened for inclusion. Pediatric and neonatal cases (defined as age < 18 years) were excluded because age-dependent infection syndromes, specimen distributions, antimicrobial testing practices, and neonatal-unit epidemiology differ substantially from adult populations; this restriction improved comparability for adult laboratory surveillance. Duplicate isolates from the same patient and specimen type within 14 days were excluded to ensure each isolate represented a single microbiological event and was used as the unit of analysis for non-susceptibility trend surveillance.

Specimens originated from blood, urine, respiratory secretions, wound and tissue samples, and sterile body fluids. Demographic and clinical data collected included patient age, sex, hospital ward, and care setting (inpatient or outpatient).

### Microbiological identification

Isolates were initially identified using the VITEK® 2 automated microbial identification system (bioMérieux, Marcy-l’Étoile, France). When species-level identification was inconclusive or discordant, MicroScan WalkAway (Beckman Coulter, Brea, CA, USA) was used as a secondary adjudication method according to routine laboratory practice. MicroScan was not treated as a molecular reference standard or assumed to be superior to VITEK 2 for Enterobacterales identification; therefore, species-level results within the *Enterobacter cloacae* complex were interpreted conservatively.

Isolates classified within the *Enterobacter cloacae* complex, including *E. hormaechei*, *E. asburiae*, *E. cloacae*, and related taxa, were analyzed collectively because routine automated systems cannot reliably resolve all members of the complex at species level [2,4–6].

In accordance with current taxonomic standards, *Enterobacter aerogenes* isolates were excluded, as this species has been reclassified as *Klebsiella aerogenes* and no longer belongs to the *Enterobacter* genus. Species assignment followed updated genomic-based nomenclature and phylogenetic standards [15].

### Antimicrobial susceptibility testing

Antimicrobial susceptibility testing (AST) was performed primarily using the VITEK® 2 Compact system (bioMérieux, Marcy-l’Étoile, France), with selective use of MicroScan WalkAway (Beckman Coulter, Brea, CA, USA) for confirmatory testing or when required. Disk diffusion (Oxoid, Thermo Fisher Scientific, Basingstoke, UK) and gradient diffusion methods (Etest; bioMérieux, Marcy-l’Étoile, France) were used selectively to resolve uncertainty in automated results, in accordance with laboratory standard operating procedures. Non-automated AST results were interpreted according to Clinical and Laboratory Standards Institute (CLSI) performance standards applicable for the corresponding study year. Results from 2012 to 2014 were interpreted using CLSI M100-S22 (2012), and subsequent isolates according to annual updates through CLSI M100, 34th Edition (2024). Historical results were not retrospectively reinterpreted using a single modern breakpoint standard; this approach preserves the routine-reporting context but may affect temporal comparability.

European Committee on Antimicrobial Susceptibility Testing (EUCAST) guidelines were consulted only when no CLSI breakpoint existed for the organism–antibiotic combination for the corresponding study year, and EUCAST disk diffusion breakpoints from the relevant annual version (v2.0–v15.0) were applied. Isolates were categorized as susceptible (S), susceptible dose-dependent (SDD), intermediate (I), or resistant (R) according to Clinical and Laboratory Standards Institute (CLSI) or EUCAST criteria valid for the corresponding study year. For analytical consistency, non-susceptible (NS) isolates were defined as those interpreted as SDD, intermediate, or resistant (SDD + I + R), based on either minimum inhibitory concentration (MIC) or disk diffusion (DD) breakpoints. For this surveillance analysis, susceptibility results were deliberately converted to a binary susceptible versus non-susceptible variable, with susceptible-dose-dependent (SDD) and intermediate results grouped with resistant. This choice reflected several considerations: a binary outcome was required for the longitudinal trend analysis; an intermediate result is clinically managed as reduced susceptibility that lies closer to resistance and does not revert to full susceptibility; the reporting laboratory does not routinely assign or report the SDD category in its standard workflow; and SDD is a comparatively recent breakpoint addition (for example, introduced for cefepime only in 2014) that was not applied consistently across the study period. Consequently, SDD and intermediate results were not reported as separate strata for individual agents, and a sensitivity analysis excluding SDD results was neither feasible nor, given routine laboratory practice, clinically informative; the non-susceptible definition was therefore applied uniformly across all agents and study years.

Difficult-to-treat resistance (DTR) was defined according to the criteria proposed by Kadri et al. [11], as non-susceptibility to all first-line agents—that is, to all tested β-lactam categories, including extended-spectrum cephalosporins, the β-lactam/β-lactamase-inhibitor combination piperacillin–tazobactam, the monobactam aztreonam, and carbapenems, together with all tested fluoroquinolones. Operationally, an isolate was classified as DTR when it was non-susceptible to every first-line agent tested among carbapenems (ertapenem, imipenem, meropenem), extended-spectrum cephalosporins (cefotaxime, ceftriaxone, ceftazidime, cefepime), piperacillin–tazobactam, aztreonam, and fluoroquinolones (ciprofloxacin, levofloxacin). To prevent classification based on a single tested agent per class, eligibility required prior testing against at least one carbapenem, at least one extended-spectrum cephalosporin, piperacillin–tazobactam, and at least one fluoroquinolone; isolates not meeting this minimum testing coverage were excluded from the DTR denominator. CRE were defined as Enterobacterales isolates demonstrating non-susceptibility to at least one carbapenem agent (imipenem, meropenem, or ertapenem), according to CLSI M100 criteria [15].

### Data analysis

Descriptive statistics were calculated to summarize isolate-level demographic and clinical-context variables relevant to microbiological surveillance. Categorical variables were expressed as frequencies and percentages, while patient age was reported as median and interquartile range (IQR) and analyzed as a continuous variable. Associations between age, organism category (*Enterobacter cloacae* complex vs. other *Enterobacter* species), and specimen source were evaluated to assess whether apparent age differences reflected specimen distribution rather than patient outcome patterns. Unadjusted models were first performed, followed by a multivariable linear regression model including organism category and specimen source entered simultaneously. Skin samples served as the reference category. Results are presented as unstandardized regression coefficients (B) with 95% confidence intervals (CI) and p-values, representing mean differences in age (years). Model fit was assessed using R^2^, and statistical significance was defined as a two-sided p-value <0.05.

The relationship between hospitalization status (inpatient vs. outpatient) and antibiotic non-susceptibility was explored using Chi square test for each antibiotic agent, unless otherwise noted. A p-value of less than 0.05 was considered statistically significant. All statistical analyses were performed using SPSS version 25.0 (IBM Corp., Armonk, NY, USA).

To evaluate temporal trends in antimicrobial non-susceptibility over the 12-year (August 2012–August 2024, comprising 13 consecutive calendar years) study period, univariate binary logistic regression models were constructed for each antimicrobial agent. In each model, year of isolate collection was entered as a continuous independent variable, and the binary susceptibility outcome (non-susceptible vs. susceptible) served as the dependent variable. Regression outputs included the regression coefficient (B), p-value, odds ratio (OR; Exp(B)), and corresponding 95% confidence intervals (CIs). An OR greater than 1 was interpreted as an increasing temporal trend in non-susceptibility, whereas an OR less than 1 indicated a decreasing trend over time. To enhance clinical interpretability, the annual percent change (APC) in the odds of non-susceptibility was calculated for each agent using the formula APC = (OR − 1) × 100, representing the average yearly change in odds derived from logistic regression models. Because the APC is derived from the odds ratio, it represents the average annual change in the odds of non-susceptibility rather than the absolute change in the resistance proportion. To address potential confounding, multivariable binary logistic regression models were additionally constructed for each agent, adjusting for care setting (outpatient, hospital ward, or intensive-care unit), specimen source, patient age, and sex, while retaining year of collection as the continuous exposure of interest; adjusted odds ratios and APCs are reported in S6 Table. Antibiotics evaluated exclusively in urine isolates were analyzed within the corresponding restricted dataset. All statistical tests were two-sided, and a p-value < 0.05 was considered statistically significant.

Additionally, two clinically significant non-susceptibility phenotypes were assessed: CRE and DTR. Isolates were assigned to these phenotypes only if all specified criteria were met. Annual proportions of CRE and DTR isolates were calculated and their trends visualized using Python version 3.11 (Python Software Foundation, Wilmington, DE, USA) and the matplotlib library version 3.7.1. For each year, the denominator for these phenotypes comprised the isolates tested for at least one agent within every required antimicrobial class in that year; isolates without any susceptibility result for an entire required class were excluded from the corresponding annual denominator. Annual numerators, denominators, and eligibility criteria for both phenotypes are reported in S7 Table.

To account for the inclusion of multiple isolates from some patients (1,319 isolates from 1,000 patients), all temporal-trend models were refitted with cluster-robust (sandwich) standard errors clustered on the patient identifier and, in a further sensitivity analysis, were restricted to the first isolate per patient. Two additional sensitivity analyses assessed temporal comparability: exclusion of the two partial calendar years (2012, sampled from August, and 2024, sampled through August); and restriction of each agent’s trend to the interval preceding the major CLSI breakpoint revision affecting that agent (fluoroquinolones, 2019; piperacillin–tazobactam, 2022; aminoglycosides, 2023) or, for cefepime, to the interval following introduction of the susceptible-dose-dependent category (2014). A timeline of AST platforms, testing panels, interpretive standards, and major breakpoint revisions across the study period is provided in S8 Table. Logistic-regression trend models with cluster-robust standard errors were implemented in Python version 3.11; other analyses used SPSS version 25.0.

Analyses for antibiotics restricted to certain specimen types such as Fosfomycin and Nitrofurantoin, which are typically reported only for urinary isolates were confined to relevant specimen groups.

### Ethical considerations

The study was conducted using retrospective, de-identified routine microbiological laboratory data with no direct patient identifiers. Data were accessed and extracted for research purposes between 17/04/2025 and 17/07/2025. The investigators did not have access to any information that could identify individual participants during or after data collection. Ethical approval was obtained from the Institutional Review Board (IRB) of Aseer Central Hospital, Ministry of Health, Saudi Arabia (IRB No. F7-2-2025). The requirement for informed consent was waived by the IRB due to the retrospective nature of the study and the use of fully de-identified data. The research was conducted in accordance with the principles of the Declaration of Helsinki (2013 revision) and applicable national research governance standards.

## Results

### Patient and isolate characteristics

During the study period (2012–2024), a total of 1,000 patients contributed 1,319 non-duplicate *Enterobacter* isolates to the analysis. The median patient age was 53.0 years (IQR, 38.0), and the cohort was predominantly male (71.8%). Most isolates were obtained from hospitalized patients, with inpatient cases accounting for 90.0% of all isolates, while 10.0% were recovered from outpatients. Among inpatient isolates, the largest proportion originated from surgical wards (48.3%), followed by medical wards (32.3%), and intensive care units (19.5%). Overall, 17.5% of isolates were associated with ICU admission.

Regarding clinical context, a history of trauma or prolonged hospitalization following road traffic accidents or burn injuries was documented in 14.6% of isolates. Chronic comorbid conditions, including diabetes mellitus and end-stage renal disease, were recorded in 3.3%, while 1.1% of isolates were associated with concurrent COVID-19 infection. For the majority of isolates (80.9%), no specific underlying condition was documented (Table 1).

**Table 1:** Demographic, clinical, and hospitalization characteristics of patients and isolates included in the *Enterobacter* study Age and sex are summarized at the patient level (n = 1,000), whereas clinical condition, care setting, hospitalization ward, and ICU admission are summarized at the isolate level (n = 1,319).

Characteristic	Total Patients (n = 1000)
Age [Median (IQR)]	53.0 (38.0)
Sex	
Male	718 (71.8%)
Female	282 (28.2%)
	**Total isolates** **(n = 1319)**
Clinical/ underlying condition	
Trauma/ long-stay after RTA or burn	193 (14.6%)
Chronic comorbidity (e.g., ESRD, DM)	44 (3.3%)
COVID-19 infection	15 (1.1%)
Other/ none recorded	1 067 (80.9%)
Care setting	
Inpatient (INP)	1187 (90.0%)
Outpatient (OPD)	132 (10.0%)
Hospitalization Ward (n = 1187)	
Medical ward	383 (32.3%)
Surgical ward	573 (48.3%)
ICU ward	231 (19.5%)
ICU admission	
ICU	231 (17.5%)
Non-ICU	1088 (82.5%)

*Note.* Age expressed as median (interquartile range, IQR). admission lasting ≥ 2 weeks, usually following major trauma such as burn injury or road-traffic accident (RTA); ESRD: End-Stage Renal Disease; COVID-19: Coronavirus Disease 2019; DM: Diabetes Mellitus; ICU: (include Intermediate + Intensive Care Unit); IQR: interquartile Range.

Additional comparative analyses of demographic and clinical characteristics between *Enterobacter cloacae* complex and other *Enterobacter* species are provided (S1 Table).

### Temporal distribution of *Enterobacter* species isolates

Between 2012 and 2024, a total of 1,319 *Enterobacter* isolates were identified, of which 1,211 (91.8%) belonged to the *Enterobacter cloacae* complex and 108 (8.2%) to other *Enterobacter* species. Across all study years, *E. cloacae* complex consistently predominated, accounting for ≥80% of isolates annually and reaching 100% in 2022. Total annual isolate counts peaked in 2019 (n = 137) and 2021 (n = 144) (Fig 1). Within the other *Enterobacter* species group (n = 108), the most frequent laboratory identifications were *Enterobacter* species not further differentiated (n = 41), the *Enterobacter agglomerans* group (n = 26), *Enterobacter gergoviae* (n = 13), *Enterobacter sakazakii* (n = 10), *Enterobacter amnigenus* (n = 7), *Enterobacter cancerogenus* (n = 6), and *Enterobacter intermedius* (n = 5); several of these taxa have since been reclassified into other genera (for example *Pantoea*, *Pluralibacter*, *Cronobacter*, *Kosakonia*, and *Lelliottia*). The full species-level distribution is provided in S11 Table.

**Fig 1:**
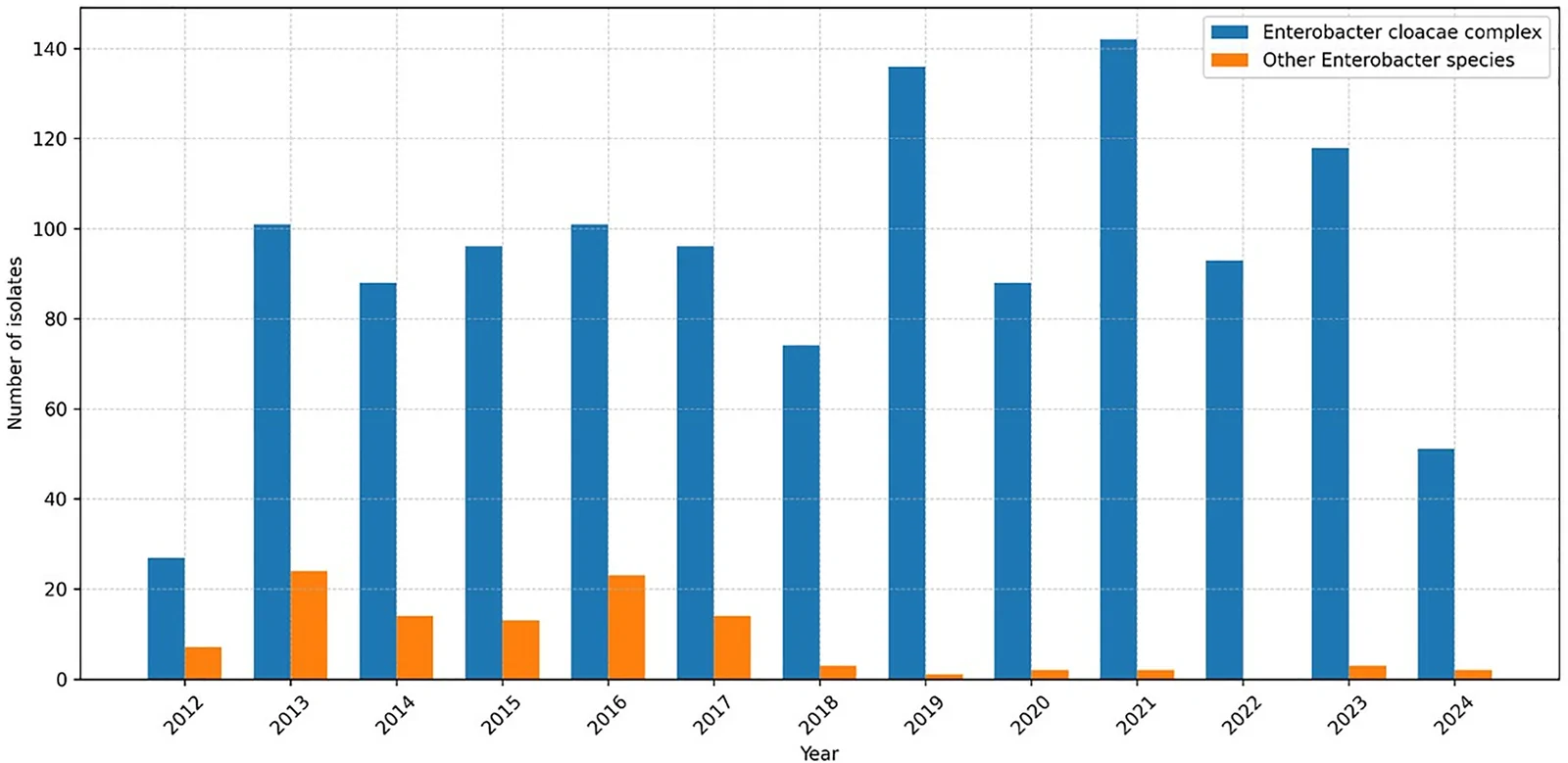
Clustered bar chart showing annual counts of *Enterobacter cloacae* complex and other *Enterobacter* species isolates collected between 2012 and 2024. Bars represent absolute isolate counts per year, highlighting the consistently higher burden of *Enterobacter cloacae* complex compared with non-*cloacae Enterobacter* species across the study period.

### Distribution of clinical specimen sources by *Enterobacter* species group

Clinical specimen sources differed between *Enterobacter cloacae* complex and other *Enterobacter* species isolates (Table 2). Overall, skin and soft tissue specimens represented the most frequent source (32.4%), followed by urine (25.5%) and respiratory specimens (15.2%). Respiratory specimens were proportionally more common among non-*E. cloacae* complex *Enterobacter* species than among *E. cloacae* complex isolates (23.1% vs. 14.5%; p = 0.017), whereas skin specimens were proportionally more frequent among *E. cloacae* complex isolates (33.2% vs. 23.1%; p = 0.032). These associations should be interpreted as descriptive source-distribution differences rather than evidence of tissue tropism or causality.

**Table 2:** Distribution of clinical specimen sources by *Enterobacter* species group (n = 1319).

Characteristic	Total samples n = 1319	*Enterobacter cloacae* Complex n = 1211	*Enterobacter* species n = 108	P-value
**Source**				
Respiratory	201/1319 (15.2%)	176/1211 (14.5%)	25/108 (23.1%)	0.017^a^
Skin samples	427/1319 (32.4%)	402/1211 (33.2%)	25/108 (23.1%)	0.032^a^
Urine	337/1319 (25.5%)	302/1211 (24.9%)	35/108 (32.4%)	0.088^a^
Blood	103/1319 (7.8%)	96/1211 (7.9%)	7/108 (6.5%)	0.592^a^
Abscess	111/1319 (8.4%)	105/1211 (8.7%)	6/108 (5.6%)	0.264^a^
Other	140/1319 (10.1%)	130/1211 (10.7%)	10/108 (9.3%)	0.633

*Note.* a- Chi Square Test.

Urinary isolates were numerically more common among other *Enterobacter* species compared with *E. cloacae* complex isolates (32.4% vs. 24.9%), although this difference did not reach statistical significance. No significant differences were observed between species groups for bloodstream, abscess, or other specimen sources (Table 2).

### Association between organism category, specimen source, and patient age

In unadjusted analysis, patients from whom non–*Enterobacter cloacae* complex species were isolated were significantly older than those with *E. cloacae* complex isolates (mean difference, + 6.9 years; p = 0.002), a finding that is visually demonstrated by the age distribution between species groups (Fig 2). When specimen source was incorporated into a multivariable linear regression model, the association between organism category and age was partially attenuated but remained statistically significant (adjusted mean difference, + 5.1 years; p = 0.016), indicating that differences in clinical source distribution explain a substantial proportion, but not all, of the observed age difference. Specimen source emerged as a major determinant of age, with urinary, bloodstream, and respiratory isolates showing the strongest independent associations with older age, whereas skin and other sources showed weaker or non-significant effects. Overall, inclusion of specimen source variables markedly improved model explanatory power (adjusted R^2^ = 0.139 vs. 0.007 in the unadjusted model), supporting a source-driven contribution to age differences across *Enterobacter* species groups (Table 3; S2 Table).

**Table 3:** Linear regression analysis of factors associated with patient age (n = 1319).

Variable	Adjusted B (95% CI)*	P-value
Organism category (Other *Enterobacter* vs *E. cloacae* complex)	5.06 (0.94–9.18)	0.016
Specimen source		
Respiratory	9.62 (6.11–13.13)	<0.001
Skin samples	Reference	—
Urine	20.14 (17.15–23.12)	<0.001
Blood	15.78 (11.29–20.26)	<0.001
Abscess	6.71 (2.35–11.06)	0.003
Other source	2.46 (−1.52 to 6.44)	0.225
Model Summary		
Model R²	0.139	—
Model p-value	<0.001	—

*Note.* *Adjusted model includes organism category and specimen source variables entered simultaneously; skin samples served as the reference category.

**Fig 2:**
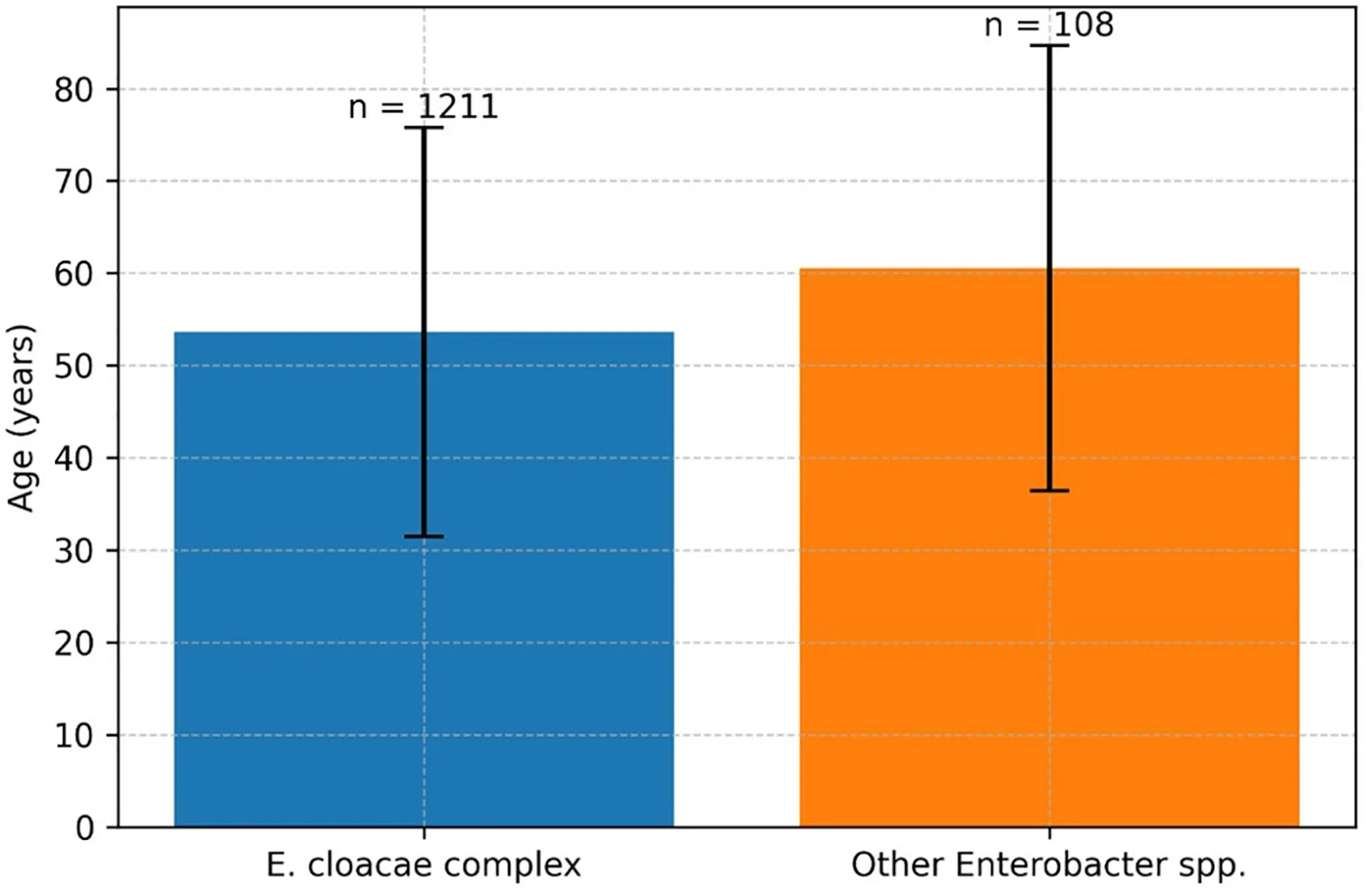
Comparison of patient age by *Enterobacter* species group. Bars represent the mean age (years) of patients with *Enterobacter cloacae* complex and other *Enterobacter* species isolates; error bars denote standard deviation (SD). Sample sizes for each group are indicated above the bars. Dashed grid lines are shown to aid visual comparison between groups.

### Comparative non-susceptibility patterns between *Enterobacter cloacae* complex and other *Enterobacter* species by antibiotic class

#### Aminoglycosides.

Non-susceptibility to aminoglycosides was generally comparable between organism groups. Amikacin showed a numerically higher non-susceptibility rate among other *Enterobacter* species than *E. cloacae* complex (21.8% vs. 15.0%), although this difference did not reach statistical significance (p = 0.071). Gentamicin demonstrated a similar pattern, with higher non-susceptibility among other *Enterobacter* species (38.2% vs. 29.3%), again approaching but not achieving statistical significance (p = 0.060). Tobramycin non-susceptibility rates were comparable between groups without significant difference (p = 0.551) (Table 4).

**Table 4:** Percentages represent non-susceptible isolates (susceptible-dose-dependent, intermediate, or resistant), interpreted according to CLSI criteria.

Family	Antibiotic	Total samples n = 1319	Organism category			P-Value
			*Enterobacter cloacae* Complex n = 1211	*Enterobacter* species n = 108	Absolute difference (%)	
Aminoglycosides	Amikacin	200/1288 (15.5%)	178/1187 (15.0%)	22/101 (21.8%)	6.8	0.071^a^
	Gentamicin	383/1275 (30.0%)	344/1173 (29.3%)	39/102 (38.2%)	8.9	0.060^a^
	Tobramycin	274/837 (32.7%)	258/782 (33.0%)	16/55 (29.1%)	3.9	0.551^a^
Penicillin’s/ cephalosporins	Aztreonam	416/605 (68.8%)	401/577 (69.5%)	15/28 (53.6%)	15.9	0.076^a^
	Cefotaxime	377/613 (61.5%)	356/569 (62.6%)	21/44 (47.7%)	14.9	0.051^a^
	Cefepime	506/1151 (44.0%)	463/1060 (43.7%)	43/91 (47.3%)	3.6	0.510^a^
	Ceftazidime	689/1171 (58.8%)	641/1072 (59.8%)	48/99 (48.5%)	11.3	0.029^a,d^
	Ceftriaxone	84/157 (53.5%)	83/153 (54.2%)	1/4 (25.0%)^e^	29.2	0.338^b^
	Cefuroxime	493/568 (86.8%)	469/530 (88.5%)	24/38 (63.2%)	25.3	<0.001^a,d^
	Piperacillin-tazobactam	449/1212 (37.0%)	429/1130 (38.0%)	20/82 (24.4%)	13.6	0.014^a,d^
Fluoroquinolones	Ciprofloxacin	469/1262 (37.2%)	418/1162 (36.0%)	51/100 (51.0%)	15.0	0.003^a^
	Levofloxacin	263/1012 (26.0%)	229/935 (24.5%)	34/77 (44.2%)	19.7	<0.001^a,d^
Carbapenems	Ertapenem	265/764 (34.7%)	256/727 (35.2%)	9/37 (24.3%)	10.9	0.175^a^
	Imipenem	229/1237 (18.5%)	210/1138 (18.5%)	19/99 (19.2%)	0.7	0.856^a^
	Meropenem	174/1173 (14.8%)	159/1107 (14.4%)	15/66 (22.7%)	8.3	0.063^a^
Urinary-specific agents	Fosfomycin^c^	12/43 (27.9%)	10/31 (32.3%)	2/12 (16.7%)^e^	15.6	0.456^b^
	Nitrofurantoin^c^	99/159 (62.3%)	85/131 (64.9%)	14/28 (50.0%)	14.9	0.140^a^
Tetracyclines	Tetracycline	78/182 (42.9%)	65/154 (42.2%)	13/28 (46.4%)	4.2	0.678^a^

*Note.* a– Chi-square test; b- Fisher’s exact test; c – for urine samples only.

Denominators vary due to differences in antibiotic testing availability across isolates and study years.

d-Indicating significance.

e-Small sample sizes should be interpreted with caution.

#### Penicillin’s and cephalosporins.

Marked differences were observed within the β-lactam class. Non-susceptibility to cefuroxime was significantly higher among *E. cloacae* complex isolates compared with other *Enterobacter* species (88.5% vs. 63.2%, p < 0.001). Similarly, ceftazidime non-susceptibility was more frequent in *E. cloacae* complex (59.8% vs. 48.5%, p = 0.029). Cefotaxime showed a borderline, non-significant difference in the same direction, with lower non-susceptibility among other *Enterobacter* species (47.7% vs. 62.6%, p = 0.051), while cefepime and ceftriaxone demonstrated no statistically significant differences between groups. Aztreonam non-susceptibility was high in both groups, with no significant interspecies difference (p = 0.076) (Table 4).

Carbapenem non-susceptibility rates were broadly comparable between organism groups. No significant differences were observed for imipenem or ertapenem. Meropenem non-susceptibility was numerically higher among other *Enterobacter* species (22.7% vs. 14.4%), although this difference did not reach statistical significance (p = 0.063).

Non-susceptibility to piperacillin–tazobactam differed significantly between groups, with *E. cloacae* complex isolates demonstrating higher non-susceptibility rates than other *Enterobacter* species (38.0% vs. 24.4%, p = 0.014).

#### Fluoroquinolones.

Fluoroquinolone non-susceptibility patterns differed substantially between organism groups. Non-susceptibility to ciprofloxacin was significantly higher among other *Enterobacter* species compared with *E. cloacae* complex (51.0% vs. 36.0%, p = 0.003). A similar and more pronounced difference was observed for levofloxacin, with other *Enterobacter* species exhibiting markedly higher non-susceptibility (44.2% vs. 24.5%, p < 0.001). These findings indicate a stronger association between non-*E. cloacae* complex species and fluoroquinolone non-susceptibility (Table 4).

#### Urinary-specific agents.

Among urinary isolates only, fosfomycin and nitrofurantoin showed no statistically significant differences in non-susceptibility between organism groups, although numerically lower rates were observed among other *Enterobacter* species for both agents (Table 4).

#### Tetracyclines.

Tetracycline non-susceptibility rates were similar between *E. cloacae* complex and other *Enterobacter* species, with no significant intergroup difference observed (Table 4).

### Antimicrobial non-susceptibility according to clinical setting (inpatient vs outpatient)

#### Aminoglycosides.

Non-susceptibility to aminoglycosides was generally comparable between inpatient and outpatient isolates. Amikacin showed similar non-susceptibility rates in inpatient and outpatient settings (15.6% vs. 14.5%), with no statistically significant difference. Tobramycin also demonstrated nearly identical non-susceptibility rates between settings. In contrast, gentamicin non-susceptibility was significantly higher among inpatient isolates compared with outpatient isolates (31.7% vs. 14.8%, p < 0.001) (S3 Table), indicating a statistically significant association between inpatient care and gentamicin non-susceptibility.

#### Penicillin’s and cephalosporins.

Marked differences were observed for several β-lactam agents. Inpatient isolates exhibited significantly higher non-susceptibility to third- and fourth-generation cephalosporins, including cefotaxime (63.9% vs. 41.5%, p < 0.001), cefepime (45.7% vs. 28.8%, p < 0.001), ceftazidime (61.4% vs. 35.3%, p < 0.001), and ceftriaxone (57.4% vs. 18.8%, p = 0.003). Non-susceptibility to cefuroxime was high in both settings without a statistically significant difference, suggesting widespread non-susceptibility independent of care setting. Aztreonam non-susceptibility was also high in both groups, with a non-significant trend toward higher rates among inpatients.

Carbapenem non-susceptibility rates were similar between inpatient and outpatient isolates. No statistically significant differences were observed for ertapenem, imipenem, or meropenem, although numerically higher non-susceptibility rates were consistently seen among inpatient isolates. These findings suggest that carbapenem non-susceptibility in *Enterobacter* spp. was not confined to inpatient isolates in this study.

A significant difference was observed for piperacillin–tazobactam, with inpatient isolates demonstrating substantially higher non-susceptibility compared with outpatient isolates (38.7% vs. 23.1%, p < 0.001). This pattern is compatible with differences in inpatient case mix, prior healthcare exposure, device use, and antimicrobial exposure, but antimicrobial consumption data were not available to directly test selection pressure.

#### Fluoroquinolones.

Fluoroquinolone non-susceptibility did not differ significantly by clinical setting. Ciprofloxacin and levofloxacin showed comparable non-susceptibility rates between inpatient and outpatient isolates, indicating that non-susceptibility to this class was prevalent across both care settings rather than confined to hospitalized patients.

#### Urinary-specific agents.

Among urine isolates only, non-susceptibility to fosfomycin and nitrofurantoin did not differ significantly between inpatient and outpatient settings. Non-susceptibility rates for both agents were comparable, but interpretation is limited by restricted urinary testing and small denominators, particularly for fosfomycin.

#### Tetracyclines.

Non-susceptibility to tetracycline was similar between inpatient and outpatient isolates, with no statistically significant difference observed. However, interpretation is limited by the relatively small number of outpatient isolates tested (S3 Table).

### Temporal trends in antimicrobial non-susceptibility across *Enterobacter* species

Logistic regression analysis demonstrated heterogeneous yet clinically significant temporal trends in antimicrobial non-susceptibility among *Enterobacter* species over the study period (Table 5). The most pronounced increases were observed among carbapenems and selected broad-spectrum β-lactams, indicating a progressive escalation in non-susceptibility burden. Non-susceptibility to ertapenem increased significantly over time (OR 1.157 per year, corresponding to an APC of +15.7%, *p* < 0.001), alongside parallel increases for imipenem (*OR 1.130; APC + 13.0%; p* < 0.001) and meropenem (OR 1.166; APC + 16.6%; *p* < 0.001). Collectively, these findings indicate a sustained and substantial rise in carbapenem non-susceptibility across *Enterobacter* species.

**Table 5:** Temporal Trends in Antimicrobial Non-Susceptibility Among *Enterobacter* Species. N denotes the number of isolates tested for each agent (the denominator). Annual percent change (APC) reflects the average annual change in the odds of non-susceptibility; full unadjusted and multivariable-adjusted estimates are provided in S6 Table.

Family	Antibiotic	Exp(B) (OR)	P-value	APC (% per year)	95% CI for Exp(B)
Aminoglycosides	Amikacin (N = 1,288)	0.942	0.009	−5.8%	0.901–0.985
	Gentamicin (N = 1,275)	0.900	<0.001	−10.0%	0.869–0.933
	Tobramycin (N = 837)	0.982	0.423	−1.8%	0.941–1.026
Penicillin’s/ cephalosporins	Aztreonam (N = 605)	1.010	0.700	+1.0%	0.961–1.062
	Cefotaxime (N = 613)	1.056	0.022	+5.6%	1.008–1.107
	Cefepime (N = 1,151)	0.906	<0.001	−9.4%	0.875–0.939
	Ceftazidime (N = 1,171)	0.971	0.083	−2.9%	0.938–1.004
	Ceftriaxone (N = 157)	0.977	0.621	−2.3%	0.890–1.072
	Cefuroxime (N = 568)	1.049	0.168	+4.9%	0.980–1.124
	Piperacillin–tazobactam (N = 1,212)	1.065	0.001	+6.5%	1.027–1.104
Fluoroquinolones	Ciprofloxacin (N = 1,262)	1.005	0.765	+0.5%	0.972–1.039
	Levofloxacin (N = 1,012)	1.075	0.001	+7.5%	1.030–1.122
Carbapenems	Ertapenem (N = 764)	1.157	<0.001	+15.7%	1.099–1.218
	Imipenem (N = 1,237)	1.130	<0.001	+13.0%	1.083–1.179
	Meropenem (N = 1,173)	1.166	<0.001	+16.6%	1.105–1.231
Urinary-specific agents	Fosfomycin†	1.040	0.711	+4.0%	0.844–1.283
	Nitrofurantoin†	1.025	0.628	+2.5%	0.928–1.132
Tetracyclines	Tetracycline	1.088	0.077	+8.8%	0.991–1.195

*Note.* APC (Annual Percent Change) calculated as (Exp(B) − 1) × 100, representing the average annual change in the odds of non-susceptibility, †;Urine isolates only.

A similar upward trend was observed for piperacillin–tazobactam, with a significant annual increase in the odds of non-susceptibility (OR 1.065; APC + 6.5%; p = 0.001), indicating an increasing odds-based trend rather than an absolute annual increase in the resistance proportion. Within the cephalosporin class, divergent temporal patterns were evident. Cefotaxime demonstrated a modest but significant increase in non-susceptibility (OR 1.056; APC + 5.6%; p = 0.022), whereas cefepime showed a marked and statistically significant decline over time (OR 0.906; APC −9.4%; p < 0.001). Other cephalosporins, including ceftazidime (OR 0.971; APC −2.9%; p = 0.083), ceftriaxone (OR 0.977; APC −2.3%; p = 0.621), and cefuroxime (OR 1.049; APC + 4.9%; p = 0.168), did not exhibit statistically significant temporal trends.

In contrast, aminoglycosides showed consistent and significant downward trends in non-susceptibility. Both amikacin (OR 0.942; APC −5.8%; *p* = 0.009) and gentamicin (OR 0.900; APC −10.0%; *p* < 0.001) demonstrated decreasing odds of non-susceptibility over time, suggesting relative preservation of susceptibility within this class. Fluoroquinolone trends were mixed: levofloxacin exhibited a significant upward trend (OR 1.075; APC + 7.5%; *p* = 0.001), whereas ciprofloxacin showed no meaningful temporal change (OR 1.005; APC + 0.5%; *p* = 0.765). No statistically significant trends were observed for aztreonam, tetracycline, tobramycin, fosfomycin, or nitrofurantoin.

After multivariable adjustment for care setting, specimen source, patient age, and sex, the direction, magnitude, and statistical significance of these temporal trends were essentially unchanged, indicating that the observed trajectories were not explained by these factors. Adjusted increases persisted for the carbapenems (ertapenem adjusted APC + 15.6%; imipenem +13.1%; meropenem +15.8%; all p < 0.001), as well as for piperacillin–tazobactam (+7.0%; p < 0.001) and levofloxacin (+6.3%; p = 0.008), whereas adjusted declines persisted for amikacin (−6.1%; p = 0.006), gentamicin (−9.4%; p < 0.001), and cefepime (−9.1%; p < 0.001); ciprofloxacin remained stable (+0.5%; p = 0.774). Full unadjusted and adjusted estimates for all agents are provided in S6 Table.

These regression-derived temporal patterns are visually corroborated by the longitudinal non-susceptibility trajectories depicted in S1 Fig, which illustrates annual trends across major antimicrobial classes for all *Enterobacter* species. The figure highlights a sustained upward trajectory in non-susceptibility to carbapenems and piperacillin–tazobactam, contrasted by relative stability or decline among aminoglycosides. Divergent cephalosporin trends are also evident, with increasing non-susceptibility to cefotaxime occurring alongside a marked decline for cefepime. Collectively, these visual trends reinforce the regression findings and underscore the heterogeneous yet clinically meaningful evolution of antimicrobial non-susceptibility across *Enterobacter* species over the study period.

These antimicrobial-specific trends occurred alongside a marked rise in clinically significant resistance phenotypes. CRE increased significantly over the study period (APC + 22.8%; 95% CI 18.1–27.6%; p < 0.001), rising from 11–15% in 2012–2016 to 40–52% in 2021–2023 (377/1,313 isolates overall; 28.7%). DTR, defined by the full Kadri criteria, was identified in 83 of 1,114 eligible isolates (7.5%) and also increased over time (APC + 16.4%; 95% CI 8.3–25.1%; p < 0.001); however, CRE rose earlier and remained high, whereas DTR rose later and remained infrequent, reaching its highest annual value only in 2023 (23/114; 20.2%), indicating a dissociation in timing and overall frequency between carbapenem non-susceptibility and loss of all first-line agents. The lower DTR value recorded in 2024 (3/49; 6.1%) derives from an incomplete calendar year (sampled through August) and is therefore not interpreted as a genuine decline. Annual numerators, denominators, and eligibility criteria for both phenotypes are provided in S7 Table. Stratified analyses showed DTR was somewhat more frequent among other *Enterobacter* species (8/80; 10.0%) than the *Enterobacter cloacae* complex (75/1,034; 7.3%) and among inpatient (79/999; 7.9%) than outpatient (4/115; 3.5%) isolates (S4 Table; Fig 3).

**Fig 3:**
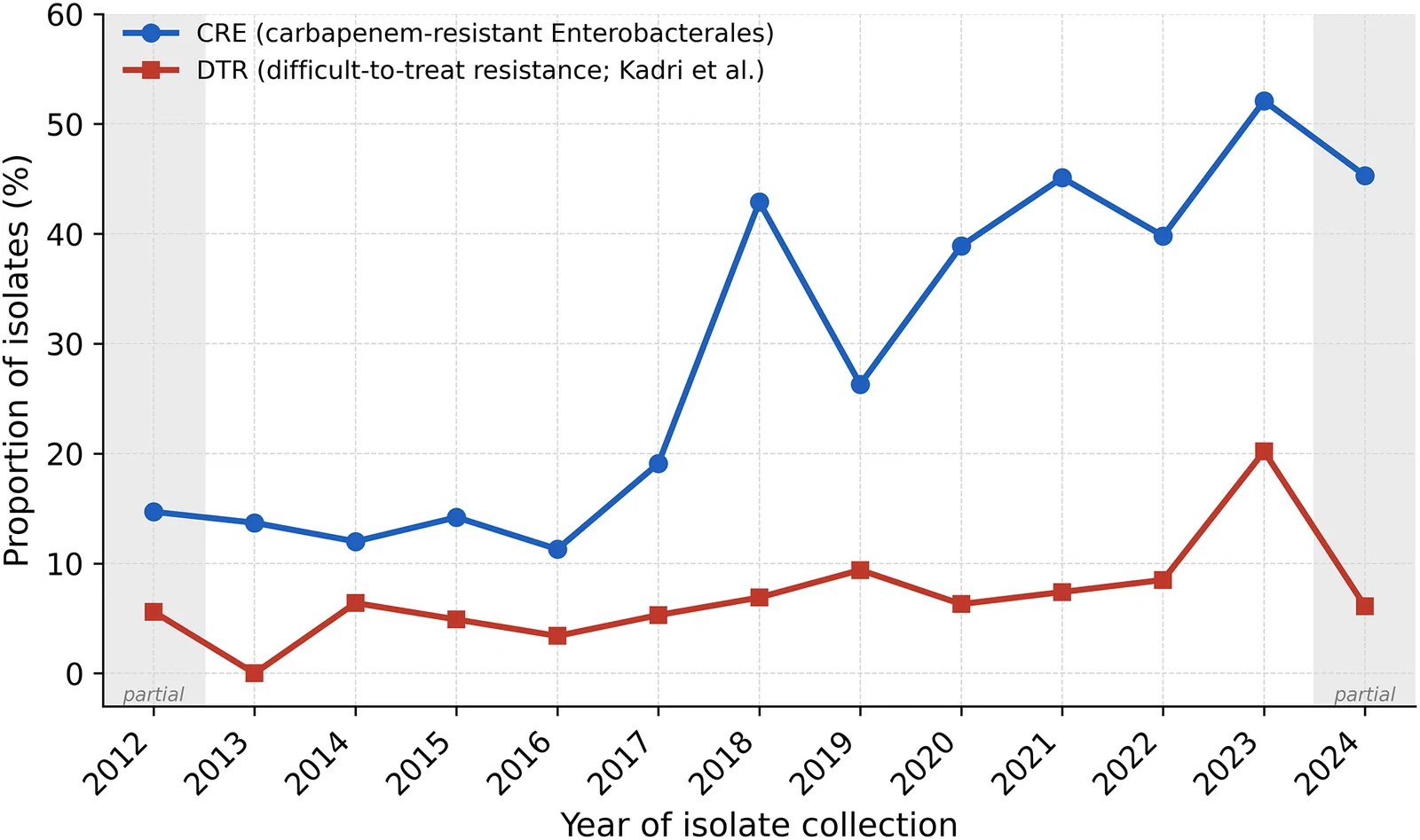
Temporal trends in carbapenem-resistant Enterobacterales (CRE) and difficult-to-treat resistance (DTR) among all *Enterobacter* species, 2012–2024. Lines show the annual proportion (%) of isolates meeting CRE (blue) and DTR (red) criteria; DTR was defined by the full criteria of Kadri et al. [11]. CRE increased from 2017 onward and remained high, whereas DTR rose later and reached its highest annual value in 2023; the first (2012) and last (2024) calendar years were incompletely sampled (shaded), so the lower 2024 DTR value should not be read as a true decline. Annual numerators, denominators, and eligibility criteria are provided in S7 Table.

Principal findings were robust in sensitivity analyses. Accounting for repeated isolates from the same patient using cluster-robust standard errors did not alter the significance of any major trend, and results were consistent when the analysis was restricted to the first isolate per patient (for example, meropenem APC + 15.2%, ertapenem +12.3%, gentamicin −10.4%, cefepime −8.8%; CRE + 22.4% and DTR + 10.1%; all p < 0.05). Excluding the two partial calendar years (2012 and 2024) left all trends materially unchanged and, for DTR, produced a stronger positive trend (APC + 20.8%; p < 0.001), indicating that the apparent 2024 DTR decline was influenced by incomplete sampling of that calendar year. In breakpoint-comparable–period analyses, the carbapenem, cefepime, and aminoglycoside trends persisted, whereas the increases in levofloxacin (APC + 7.5% overall vs + 3.6% restricted to 2012–2018; p = 0.57) and piperacillin–tazobactam (+6.5% vs + 3.0% restricted to 2012–2021; p = 0.26) were attenuated and no longer significant once analysis was confined to the period preceding the corresponding CLSI breakpoint revision. Full sensitivity-analysis results are provided in S9 and S10 Tables.

### Temporal trends in antimicrobial non-susceptibility within the *Enterobacter cloacae* complex

In analysis restricted to the *Enterobacter cloacae* complex, logistic regression identified significant temporal increases in non-susceptibility to carbapenems. Ertapenem (OR 1.160, *P* < 0.001; APC + 16.0%), imipenem (OR 1.133, *P* < 0.001; APC + 13.3%), and meropenem (OR 1.190, *P* < 0.001; APC + 19.0%) demonstrated the most pronounced upward trends. Increasing non-susceptibility was also observed for levofloxacin (OR 1.120, *P* < 0.001; APC + 12.0%), piperacillin–tazobactam (OR 1.064, *P* = 0.001; APC + 6.4%), and tetracycline (OR 1.115, *P* = 0.034; APC + 11.5%).

Conversely, significant declining trends were noted for cefepime (OR 0.905, *P* < 0.001; APC −9.5%), gentamicin (OR 0.910, *P* < 0.001; APC −9.0%), and ceftazidime (OR 0.960, *P* = 0.026; APC −4.0%). No statistically significant temporal changes were observed for aztreonam, ceftriaxone, cefuroxime, ciprofloxacin, tobramycin, fosfomycin, or nitrofurantoin (*P* > 0.05). Overall, *Enterobacter cloacae* complex-specific trends mirrored genus-level findings but with more marked carbapenem escalation, highlighting the central role of this complex in driving non-susceptibility dynamics (S5 Table).

## Discussion

This long-term laboratory-based surveillance study describes antibiotic-specific temporal trends in phenotypic antimicrobial non-susceptibility among *Enterobacter* species isolated in a tertiary-care hospital over a 12-year period. Rather than demonstrating a uniform or linear progression of non-susceptibility, the findings reveal heterogeneous and agent-specific patterns that vary by antimicrobial class and clinical context. These observations support sustained laboratory surveillance while emphasizing that the study did not evaluate antimicrobial prescribing, molecular mechanisms, transmission dynamics, or clinical outcomes.

### Clinical significance and taxonomic complexity of *Enterobacter* species

In the present cohort, non-susceptibility dynamics were largely shaped by the *Enterobacter cloacae* complex, which constituted the predominant clinical subgroup. This is consistent with prior molecular and taxonomic studies showing that the *Enterobacter cloacae* complex is a heterogeneous assemblage of related species with species-level differences in antimicrobial non-susceptibility profiles and epidemiological behavior [2,4–6]. Because the present study relied on routine automated phenotypic identification, non-susceptibility findings were interpreted at the complex level. This approach reflects routine laboratory surveillance practice, but it may obscure species-level variation and limits novelty compared with molecular or genome-based studies.

Accordingly, the results should be viewed as complex-level phenotypic surveillance rather than species-resolved molecular epidemiology. Future studies incorporating MALDI-TOF confirmation, sequence-based identification, or whole-genome sequencing would be needed to define species-level drivers, clonal relationships, and resistance mechanisms within the *Enterobacter cloacae* complex [5,6].

### Divergent temporal trends across key antimicrobial classes

A central finding of this study is the presence of divergent temporal non-susceptibility trajectories across antimicrobial classes, rather than a uniform increase in non-susceptibility. Aminoglycoside non-susceptibility declined significantly over the study period, with both amikacin and gentamicin demonstrating marked annual decreases in the odds of non-susceptibility. These findings are compatible with changing clinical use patterns or stewardship practices, but antimicrobial consumption data were not available; therefore, causal explanations based on prescribing patterns remain speculative.

In parallel, cefepime non-susceptibility declined significantly over time, a clinically relevant observation given the use of cefepime as a carbapenem-sparing option for AmpC-producing Enterobacterales [21,23]. However, this decline must be interpreted in light of evolving CLSI breakpoints, the susceptible-dose-dependent category, and potential changes in testing panels or reporting practices over the study period. Therefore, the observed decline may partly reflect interpretive and laboratory factors rather than a true biological reduction in cefepime non-susceptibility. More specifically, the cefepime breakpoints were lowered and the susceptible-dose-dependent category introduced in the 2014 CLSI revision (M100-S24); nevertheless, in a sensitivity analysis restricted to 2014–2024, the decline persisted (APC −10.7%; p < 0.001), indicating that this trend is not solely an artifact of that reclassification.

Fluoroquinolone non-susceptibility demonstrated intra-class divergence, with levofloxacin non-susceptibility increasing significantly while ciprofloxacin non-susceptibility remained relatively stable. These divergent patterns should be interpreted as antibiotic-specific surveillance observations. Without antimicrobial consumption data, molecular quinolone-resistance testing, or indication-level prescribing data, the study cannot determine whether they reflect differential selection pressure, testing practices, isolate mix, or resistance mechanisms. In addition, CLSI lowered the ciprofloxacin and levofloxacin breakpoints for Enterobacterales in 2019 (M100-Ed29); when the levofloxacin trend was restricted to the pre-2019 period, the increase was attenuated and no longer statistically significant (APC + 3.6%; p = 0.57), indicating that part of the apparent rise may reflect this interpretive change rather than a purely biological increase.

### Impact of care setting on non-susceptibility profiles

This study further demonstrates a care-setting association, with inpatient *Enterobacter* species isolates exhibiting higher non-susceptibility rates to gentamicin, multiple cephalosporins, and piperacillin–tazobactam compared with outpatient isolates. This pattern may reflect differences in case mix, healthcare exposure, device utilization, and antimicrobial exposure between inpatient and outpatient settings [3,14,23]. However, because antimicrobial consumption and patient-level treatment data were unavailable, these findings should be interpreted as epidemiological associations rather than direct evidence of prescribing-driven selection pressure.

### Age–specimen–species interactions in *Enterobacter* species epidemiology

Regression analyses showed that older patient age was associated with isolation of non-*Enterobacter cloacae* complex species, but specimen source was the more direct explanatory variable. Inclusion of specimen source substantially improved model fit and attenuated the organism-age association, indicating that the observed age difference was partly driven by the distribution of urine, respiratory, blood, and skin specimens rather than by age alone.

This finding should therefore be interpreted cautiously. Older adults may contribute different specimen types because of comorbidity, device use, urinary catheterization, aspiration risk, or healthcare exposure, and these clinical-context factors may shape which *Enterobacter* groups are recovered. The data do not establish age as a biological driver of species distribution; instead, they suggest that age, specimen source, and healthcare exposure are interrelated markers of the clinical context in which *Enterobacter* isolates are detected [6,18,24].

### Carbapenem non-susceptibility and its dissociation from DTR

Carbapenem non-susceptibility in *Enterobacter* species represents a critical escalation in non-susceptibility severity and is increasingly reported across Saudi Arabia and the broader region [10,17,19]. The present findings add longitudinal institutional data showing increasing odds of non-susceptibility to ertapenem, imipenem, and meropenem, supporting the clinical and public health relevance of ongoing CRE surveillance.

One of the most notable observations in this study is the dissociation between CRE and DTR. This dissociation rests principally on the different timing and the different overall frequency of the two phenotypes: CRE prevalence increased earlier and reached a high overall level, whereas DTR rose later and remained comparatively infrequent throughout, reaching its highest annual value only in 2023. This divergence indicates that carbapenem non-susceptibility alone does not necessarily equate to complete loss of first-line treatment options. Because antimicrobial consumption data were not available, the study cannot determine whether increasing carbapenem exposure contributed to these trends; such explanations remain hypotheses for future pharmacoepidemiological analyses. Quantitatively, CRE increased earlier and remained high (28.7% overall; APC + 22.8%), whereas DTR remained infrequent (7.5% of eligible isolates; APC + 16.4%) and reached its highest annual value in 2023 (20.2%). A lower DTR value was recorded in 2024, but because 2024 was a partial calendar year (sampled through August) it is not used as evidence of dissociation; in a sensitivity analysis excluding the partial years (2012 and 2024), the DTR trend was uniformly increasing and in fact stronger (APC + 20.8%; p < 0.001). The dissociation reported here therefore rests on the different timing and overall frequency of CRE and DTR rather than on the 2024 observation.

Despite a sustained increase in carbapenem non-susceptibility, DTR remained comparatively infrequent throughout and rose later, with its highest annual value in 2023. This pattern suggests that antibiotic class-specific non-susceptibility profiles, testing denominators, and clinical management practices may influence DTR dynamics beyond carbapenem non-susceptibility alone [11,21,23]. These findings underscore the added value of DTR as a clinically meaningful non-susceptibility metric, while also highlighting the importance of transparent denominator reporting when applying this phenotype to routine surveillance data [11]. Annual numerators, denominators, and eligibility criteria for CRE and DTR are provided in S7 Table.

### Stewardship, surveillance, and methodological considerations

International guidelines recognize several clinically important *Enterobacter* species, particularly members of the *Enterobacter cloacae* complex, as being at moderate to high risk for clinically significant AmpC production, necessitating cautious interpretation of susceptibility results and stewardship-informed antibiotic selection [23]. Both CLSI and EUCAST emphasize selective susceptibility reporting strategies, including suppression of clinically irrelevant agents, to reduce unnecessary broad-spectrum antibiotic use and to align laboratory reporting with clinical relevance [15,16].

The World Health Organization has identified antimicrobial non-susceptibility in Gram-negative bacteria, particularly non-susceptibility to third-generation cephalosporins and carbapenems, as a major global health threat, while WHO surveillance data highlight substantial gaps in non-susceptibility monitoring that disproportionately affect low- and middle-income regions [13,14]. In this context, long-term, institution-level studies such as the present analysis generate locally relevant data that may inform empirical therapy, antimicrobial stewardship policy, and infection prevention planning, provided they are interpreted alongside local prescribing and molecular epidemiology data when available.

This study has several limitations. Its retrospective, single-center design may limit generalizability to other settings. Non-susceptibility characterization relied on routine phenotypic susceptibility testing, and no molecular or genomic analyses were performed; therefore, non-susceptibility mechanisms, clonal relationships, and transmission dynamics could not be assessed. In addition, isolates were tested using evolving laboratory panels and interpreted with contemporaneous CLSI breakpoints that were revised over the study period; some apparent temporal trends, most notably the decline in cefepime non-susceptibility, which coincided with the introduction of the susceptible-dose-dependent category, may therefore partly reflect changes in interpretive criteria and reporting practices rather than true changes in the underlying organisms. Susceptibility testing was not performed uniformly for every agent, so denominators varied by antimicrobial and by year, and agents tested in only a restricted subset of isolates (for example, fosfomycin and nitrofurantoin in urinary isolates, or ceftriaxone and tetracycline) should be interpreted with corresponding caution. Species-level identification relied on automated phenotypic systems (VITEK 2 and MicroScan), and MicroScan confirmation was used as routine secondary adjudication rather than as a definitive molecular reference standard; individual species within the *Enterobacter cloacae* complex therefore could not be resolved reliably, and species-level differences may have been obscured. Antimicrobial utilization data were not available, precluding direct evaluation of associations between prescribing patterns and non-susceptibility trends. Nevertheless, the extended study period and routine laboratory methodology provide surveillance data relevant to tertiary-care settings. Because some patients contributed more than one isolate (1,319 isolates from 1,000 patients), temporal models may be subject to within-patient correlation; however, the principal findings were unchanged after cluster-robust adjustment and in analyses restricted to the first isolate per patient. The first and last calendar years were incompletely sampled (August–December 2012 and January–August 2024), which may bias year-specific estimates; sensitivity analyses excluding these partial years did not alter the overall conclusions. Susceptibility results were analyzed as a binary susceptible versus non-susceptible variable, with susceptible-dose-dependent and intermediate results grouped with resistant, because the reporting laboratory does not routinely apply the susceptible-dose-dependent category, because an intermediate result is clinically closer to resistance and is not reversible, and because susceptible-dose-dependent breakpoints were introduced only recently; susceptible-dose-dependent results (including for cefepime) therefore could not be analyzed as a separate stratum. Finally, because interpretive breakpoints and testing panels evolved over the study period (summarized in S8 Table), some apparent trends—particularly the increases in levofloxacin and piperacillin–tazobactam non-susceptibility—may partly reflect breakpoint revisions rather than true biological change, as indicated by breakpoint-comparable–period analyses (S9 Table).

## Conclusions

In conclusion, this long-term institutional analysis demonstrates that antimicrobial non-susceptibility patterns among *Enterobacter* species are heterogeneous, context-dependent, and temporally dynamic rather than uniformly progressive. Declining non-susceptibility to aminoglycosides and cefepime, divergent fluoroquinolone trends, care-setting-associated non-susceptibility patterns, and the dissociation between CRE and DTR in timing and overall frequency collectively underscore the complexity of phenotypic non-susceptibility surveillance within this genus. The apparent increases in levofloxacin and piperacillin–tazobactam non-susceptibility were attenuated to non-significance in breakpoint-comparable–period analyses and should not be regarded as unequivocal biological increases. Although multiple *Enterobacter* species were included, these temporal dynamics were predominantly driven by the *Enterobacter cloacae* complex as the principal clinical subgroup. Incorporating clinically meaningful phenotypes such as DTR enhances the interpretability of longitudinal laboratory surveillance data and provides a more nuanced framework for antimicrobial stewardship and laboratory reporting in tertiary-care settings.

## Supporting information

S1 Table Comparison of demographic, clinical, and hospitalization characteristics between *Enterobacter cloacae Complex* and Other *Enterobacter Species* isolates. (DOCX)

S2 Table Unadjusted association between *Enterobacter species* group and patient age (n = 1319). (DOCX)

S3 Table Antimicrobial non-susceptibility by clinical setting (inpatients vs outpatients) among *Enterobacter* isolates. (DOCX)

S1 Fig Temporal trends in antimicrobial non-susceptibility among *Enterobacter* species. Annual non-susceptibility rates (%) for selected antimicrobial agents among all *Enterobacter* isolates (n = 1,319) from 2012–2024 are shown. Non-susceptibility includes isolates categorized as susceptible-dose-dependent, intermediate, or resistant according to contemporaneous CLSI breakpoints. Denominators vary by agent and year due to differences in testing availability.
(TIF)

S4 Table Distribution of difficult-to-treat resistance (DTR) among *Enterobacter* isolates stratified by organism category and care setting. (DOCX)

S5 Table Temporal trends in antimicrobial non-susceptibility among *Enterobacter cloacae* complex isolates. (DOCX)

S6 Table Unadjusted and multivariable-adjusted temporal trends in antimicrobial non-susceptibility among *Enterobacter* species. Adjusted models include care setting (outpatient, hospital ward, or intensive-care unit), specimen source, patient age, and sex, with year of collection as the continuous exposure; odds ratios, annual percent change (APC), 95% confidence intervals, and the number of isolates tested per agent are reported.
(DOCX)

S7 Table Annual numerators, denominators, and eligibility criteria for carbapenem-resistant Enterobacterales (CRE) and difficult-to-treat resistance (DTR), 2012–2024. (DOCX)

S8 Table Timeline of antimicrobial susceptibility testing platforms, testing panels, interpretive standards, and major CLSI/EUCAST breakpoint revisions, 2012–2024. (DOCX)

S9 Table Partial-year and breakpoint-comparable–period sensitivity analyses of temporal non-susceptibility trends. (DOCX)

S10 Table Patient-level cluster-robust and first-isolate-per-patient sensitivity analyses. (DOCX)

S11 Table Species-level distribution within the other Enterobacter species group (n = 108). (DOCX)

S1 Dataset De-identified isolate-level dataset and accompanying data dictionary. (XLSX)
