# Supplementary material for: Laboratory-based surveillance of antimicrobial non-susceptibility in Enterobacter species over 12 years at a tertiary-care hospital in Saudi Arabia
Source: PLoS One. 2026 Oct 16;21(10):e0358963. doi: 10.1371/journal.pone.0358963 (PMC13674761; doi:10.1371/journal.pone.0358963)
Supplement: Supplementary file 1 [file pone.0358963.s001.docx]

**S1 Table.** Comparison of Demographic, Clinical, and Hospitalization Characteristics Between *Enterobacter cloacae Complex* and Other *Enterobacter Species* Isolates

| Characteristic | *Enterobacter cloacae Complex*  n = 1211 | *Enterobacter species*  n = 108 | P-value | Cramer’s V | CI 95% of V |
| --- | --- | --- | --- | --- | --- |
| Age [Median (IQR)] | 53.0 (39.0) | 63.5 (42.0) | 0.003^a^ | - | - |
| Sex |  |  |  |  |  |
| **Male** | 865/1211 (71.4%) | 71/108 (65.7%) | 0.212^b^ | 0.034 | 0.000 – 0.088 |
| **Female** | 346/1211 (28.6%) | 37/108 (34.3%) |  |  |  |
| Clinical / underlying condition |  |  |  |  |  |
| Trauma / long-stay after RTA or burn | 117/1211 (14.6%) | 16/108 (14.8%) | 0.955^b^ | 0.002 | 0.000 – 0.029 |
| Chronic comorbidity (e.g., ESRD, DM) | 41/1211 (3.4%) | 3/108 (2.8%) | 1.000^c^ | 0.009 | 0.000 – 0.061 |
| COVID-19 infection | 14/1211 (1.2%) | 1/108 (0.9%) | 1.000^c^ | 0.006 | 0.000 – 0.054 |
| Other / none recorded | 979/1211 (80.8%) | 88/108 (81.5%) | 0.871^b^ | 0.004 | 0.000 – 0.050 |
| Setting |  |  |  |  |  |
| Inpatient (INP) | 1091/1211 (90.1%) | 96/108 (88.9%) | 0.690^b^ | 0.011 | 0.000 – 0.063 |
| Outpatient (OPD) | 120/1211 (9.9%) | 12/108 (11.1%) |  |  |  |
| Hospitalization Ward |  |  |  |  |  |
| Medical ward | 354/1211 (29.2%) | 29/108 (26.9%) | 0.602^b^ | 0.014 | 0.000 – 0.067 |
| Surgical ward | 530/1211 (43.8%) | 43/108 (39.8%) | 0.427^b^ | 0.022 | 0.000 – 0.080 |
| ICU ward | 207/1211 (17.1%) | 24/108 (22.2%) | 0.179^b^ | 0.037 | 0.000 – 0.091 |
| ICU admission |  |  |  |  |  |
| ICU | 207/1211 (17.1%) | 24/108 (22.2%) | 0.179^b^ | 0.037 | 0.000 – 0.091 |
| Non-ICU | 1004/1211 (82.9%) | 84/108 (77.8%) |  |  |  |
| *Note.* a- Mann-Whitney U test; b- Chi-Square test; c– Fisher exact test; RTA: Road Traffic Accident; ESRD: End-Stage Renal Disease; COVID-19: Coronavirus Disease 2019; DM: Diabetes Mellitus; ICU: (include Intermediate + Intensive Care Unit). | | | | | |
