# Supplementary material for: Laboratory-based surveillance of antimicrobial non-susceptibility in Enterobacter species over 12 years at a tertiary-care hospital in Saudi Arabia
Source: PLoS One. 2026 Oct 16;21(10):e0358963. doi: 10.1371/journal.pone.0358963 (PMC13674761; doi:10.1371/journal.pone.0358963)
Supplement: Supplementary file 2 [file pone.0358963.s002.docx]

**S2 Table.** Unadjusted association between Enterobacter species group and patient age (n = 1319)

| Variable | Unadjusted B (95% CI) | P-value |
| --- | --- | --- |
| Organism category (Other *Enterobacter* vs *E. cloacae* complex) | 6.92 (2.52–11.32) | 0.002 |
| Model summary |  |  |
| Model R² | 0.007 | - |
| Model p-value | 0.002 | - |
| *Note.* Unstandardized regression coefficients (B) represent mean difference in age (years). | | |
