# Supplementary material for: Laboratory-based surveillance of antimicrobial non-susceptibility in Enterobacter species over 12 years at a tertiary-care hospital in Saudi Arabia
Source: PLoS One. 2026 Oct 16;21(10):e0358963. doi: 10.1371/journal.pone.0358963 (PMC13674761; doi:10.1371/journal.pone.0358963)
Supplement: Supplementary file 3 [file pone.0358963.s003.docx]

**S3 Table.** Antimicrobial non-susceptibility by clinical setting (inpatients vs outpatients) among Enterobacter isolates. Absolute difference is calculated as the inpatient percentage minus the outpatient percentage.

| Family | Antibiotic | Total samples  n = 1319 | Setting | | Absolute difference (%) | P-Value |
| --- | --- | --- | --- | --- | --- | --- |
|  |  |  | Inpatient  n = 1187 | Outpatient  n = 132 | ‍ |  |
| Aminoglycosides | Amikacin | 200/1288 (15.5%) | 181/1157 (15.6%) | 19/131 (14.5%) | 1.1 | 0.733^a^ |
|  | Gentamicin | 383/1275 (30.0%) | 364/1147 (31.7%) | 19/128 (14.8%) | 16.9 | <0.001^a^ |
|  | Tobramycin | 274/837 (32.7%) | 246/747 (32.9%) | 28/90 (31.1%) | 1.8 | 0.728^a^ |
| Penicillin’s / cephalosporins | Aztreonam | 416/605 (68.8%) | 377/539 (69.9%) | 39/66 (59.1%) | 10.8 | 0.073^a^ |
|  | Cefotaxime | 377/613 (61.5%) | 350/548 (63.9%) | 27/65 (41.5%) | 22.4 | <0.001^a^ |
|  | Cefepime | 506/1151 (44.0%) | 472/1033 (45.7%) | 34/118 (28.8%) | 16.9 | <0.001^a^ |
|  | Ceftazidime | 689/1171 (58.8%) | 648/1055 (61.4%) | 41/116 (35.3%) | 26.1 | <0.001^a^ |
|  | Ceftriaxone | 84/157 (53.5%) | 81/141 (57.4%) | 3/16 (18.8%) | 38.6 | 0.003^a^ |
|  | Cefuroxime | 493/568 (86.8%) | 444/509 (87.2%) | 49/59 (83.1%) | 4.1 | 0.369 ^a^ |
|  | Piperacillin-Tazobactam | 449/1212 (37.0%) | 419/1082 (38.7%) | 30/130 (23.1%) | 15.6 | <0.001^a^ |
| Fluoroquinolones | Ciprofloxacin | 469/1262 (37.2%) | 429/1132 (37.9%) | 40/130 (30.8%) | 7.1 | 0.111^a^ |
|  | Levofloxacin | 263/1012 (26.0%) | 229/905 (25.3%) | 34/107 (31.8%) | 6.5 | 0.149 ^a^ |
| Carbapenems | Ertapenem | 265/764 (34.7%) | 239/672 (35.6%) | 26/92 (28.3%) | 7.3 | 0.167 ^a^ |
|  | Imipenem | 229/1237 (18.5%) | 207/1111 (18.6%) | 22/126 (17.5%) | 1.1 | 0.748 ^a^ |
|  | Meropenem | 174/1173 (14.8%) | 159/1047 (15.2%) | 15/126 (11.9%) | 3.3 | 0.328 ^a^ |
| Urinary-specific agents | †Fosfomycin | 12/43 (27.9%) | 10/37 (27.0%) | 2/6 (33.3%) | 6.3 | 1.000^b^ |
|  | †Nitrofurantoin | 99/159 (62.3%) | 79/126 (62.7%) | 20/33 (60.6%) | 2.1 | 0.825 ^a^ |
| Tetracyclines | Tetracycline | 78/182 (42.9%) | 75/173 (43.4%) | 3/9 (33.3%) | 10.1 | 0.734^b^ |
| *Note.* a– Chi-square test; b- Fisher's exact test; † – urine isolates only. Denominators vary by antimicrobial agent because testing availability differed across isolates and study years. | | | | | | |
