# Supplementary material for: Laboratory-based surveillance of antimicrobial non-susceptibility in Enterobacter species over 12 years at a tertiary-care hospital in Saudi Arabia
Source: PLoS One. 2026 Oct 16;21(10):e0358963. doi: 10.1371/journal.pone.0358963 (PMC13674761; doi:10.1371/journal.pone.0358963)
Supplement: Supplementary file 4 [file pone.0358963.s005.docx]

**S4 Table.** Distribution of difficult-to-treat resistance (DTR) among *Enterobacter* isolates stratified by organism category and care setting DTR was defined using the full Kadri et al. criteria; denominators are eligible isolates tested for at least one carbapenem, at least one extended-spectrum cephalosporin, piperacillin–tazobactam, and at least one fluoroquinolone.

| Organism category | Care setting | Eligible isolates (n) | DTR, n (%) | Non-DTR, n (%) |
| --- | --- | --- | --- | --- |
| *E. cloacae complex* | Inpatients | 929 | 71 (7.6%) | 858 (92.4%) |
| *E. cloacae complex* | Outpatients | 105 | 4 (3.8%) | 101 (96.2%) |
| *E. cloacae complex* | All | 1034 | 75 (7.3%) | 959 (92.7%) |
| *Other Enterobacter species* | Inpatients | 70 | 8 (11.4%) | 62 (88.6%) |
| *Other Enterobacter species* | Outpatients | 10 | 0 (0.0%) | 10 (100.0%) |
| *Other Enterobacter species* | All | 80 | 8 (10.0%) | 72 (90.0%) |
| All isolates | Inpatients | 999 | 79 (7.9%) | 920 (92.1%) |
| All isolates | Outpatients | 115 | 4 (3.5%) | 111 (96.5%) |
| All isolates | All | 1114 | 83 (7.5%) | 1031 (92.5%) |
