# Supplementary material for: Laboratory-based surveillance of antimicrobial non-susceptibility in Enterobacter species over 12 years at a tertiary-care hospital in Saudi Arabia
Source: PLoS One. 2026 Oct 16;21(10):e0358963. doi: 10.1371/journal.pone.0358963 (PMC13674761; doi:10.1371/journal.pone.0358963)
Supplement: Supplementary file 5 [file pone.0358963.s006.docx]

**S5 Table.** Temporal Trends in Antimicrobial Non-Susceptibility Among *Enterobacter cloacae* Complex Isolates

| Family | Antibiotic | Exp(B) (OR) | P-value | APC (% per year)* | 95% CI for Exp(B) |
| --- | --- | --- | --- | --- | --- |
| Aminoglycosides | Amikacin | 0.957 | 0.065 | −4.3% | 0.913 – 1.003 |
|  | Gentamicin | 0.910 | <0.001 | −9.0% | 0.876 – 0.945 |
|  | Tobramycin | 0.990 | 0.682 | −1.0% | 0.946 – 1.037 |
| Penicillin’s / cephalosporins | Aztreonam | 1.016 | 0.557 | +1.6% | 0.964 – 1.071 |
|  | Cefotaxime | 1.046 | 0.079 | +4.6% | 0.995 – 1.099 |
|  | Cefepime | 0.905 | <0.001 | −9.5% | 0.872 – 0.939 |
|  | Ceftazidime | 0.960 | 0.026 | −4.0% | 0.926 – 0.995 |
|  | Ceftriaxone | 0.970 | 0.525 | −3.0% | 0.881 – 1.067 |
|  | Cefuroxime | 1.028 | 0.483 | +2.8% | 0.951 – 1.111 |
|  | Piperacillin–tazobactam | 1.064 | 0.001 | +6.4% | 1.024 – 1.104 |
| Fluoroquinolones | Ciprofloxacin | 1.023 | 0.201 | +2.3% | 0.988 – 1.060 |
|  | Levofloxacin | 1.120 | <0.001 | +12.0% | 1.068 – 1.174 |
| Carbapenems | Ertapenem | 1.160 | <0.001 | +16.0% | 1.098 – 1.225 |
|  | Imipenem | 1.133 | <0.001 | +13.3% | 1.082 – 1.186 |
|  | Meropenem | 1.190 | <0.001 | +19.0% | 1.123 – 1.262 |
| Urinary-specific agents | Fosfomycin† | 1.061 | 0.591 | +6.1% | 0.854 – 1.319 |
|  | Nitrofurantoin† | 1.021 | 0.709 | +2.1% | 0.916 – 1.137 |
| Tetracyclines | Tetracycline | 1.115 | 0.034 | +11.5% | 1.008 – 1.233 |
| *Note.* APC (Annual Percent Change) calculated as (Exp(B) − 1) × 100, representing the average annual change in the odds of non-susceptibility; † – urine isolates only. | | | | | |
