# Supplementary material for: Laboratory-based surveillance of antimicrobial non-susceptibility in Enterobacter species over 12 years at a tertiary-care hospital in Saudi Arabia
Source: PLoS One. 2026 Oct 16;21(10):e0358963. doi: 10.1371/journal.pone.0358963 (PMC13674761; doi:10.1371/journal.pone.0358963)
Supplement: Supplementary file 6 [file pone.0358963.s007.docx]

**S6 Table.** Unadjusted and multivariable-adjusted temporal trends in antimicrobial non-susceptibility among Enterobacter species (all isolates, 2012–2024).

| Family | Antibiotic | N tested | Unadjusted OR (95% CI) | Unadjusted APC | Unadjusted P | Adjusted OR (95% CI) | Adjusted APC | Adjusted P |
| --- | --- | --- | --- | --- | --- | --- | --- | --- |
| Aminoglycosides | Amikacin | 1288 | 0.942 (0.901–0.985) | −5.8% | 0.009 | 0.939 (0.898–0.983) | −6.1% | 0.006 |
|  | Gentamicin | 1275 | 0.900 (0.869–0.933) | −10.0% | <0.001 | 0.906 (0.874–0.940) | −9.4% | <0.001 |
|  | Tobramycin | 837 | 0.982 (0.941–1.026) | −1.8% | 0.423 | 0.979 (0.936–1.024) | −2.1% | 0.345 |
| Penicillin’s / cephalosporins | Cefuroxime | 568 | 1.049 (0.980–1.124) | +4.9% | 0.168 | 1.038 (0.965–1.117) | +3.8% | 0.311 |
|  | Cefotaxime | 613 | 1.056 (1.008–1.107) | +5.6% | 0.022 | 1.064 (1.013–1.116) | +6.4% | 0.012 |
|  | Ceftriaxone | 157 | 0.977 (0.890–1.072) | −2.3% | 0.621 | 0.978 (0.882–1.084) | −2.2% | 0.670 |
|  | Ceftazidime | 1171 | 0.971 (0.938–1.004) | −2.9% | 0.083 | 0.978 (0.945–1.013) | −2.2% | 0.211 |
|  | Cefepime | 1151 | 0.906 (0.875–0.939) | −9.4% | <0.001 | 0.909 (0.876–0.942) | −9.1% | <0.001 |
|  | Aztreonam | 605 | 1.010 (0.961–1.062) | +1.0% | 0.700 | 1.011 (0.960–1.065) | +1.1% | 0.670 |
|  | Piperacillin–tazobactam | 1212 | 1.065 (1.027–1.104) | +6.5% | <0.001 | 1.070 (1.032–1.110) | +7.0% | <0.001 |
| Carbapenems | Ertapenem | 764 | 1.157 (1.099–1.218) | +15.7% | <0.001 | 1.156 (1.097–1.218) | +15.6% | <0.001 |
|  | Imipenem | 1237 | 1.130 (1.083–1.179) | +13.0% | <0.001 | 1.131 (1.083–1.181) | +13.1% | <0.001 |
|  | Meropenem | 1173 | 1.166 (1.105–1.231) | +16.6% | <0.001 | 1.158 (1.096–1.223) | +15.8% | <0.001 |
| Fluoroquinolones | Ciprofloxacin | 1262 | 1.005 (0.972–1.039) | +0.5% | 0.765 | 1.005 (0.971–1.041) | +0.5% | 0.774 |
|  | Levofloxacin | 1012 | 1.075 (1.030–1.122) | +7.5% | 0.001 | 1.063 (1.016–1.111) | +6.3% | 0.008 |
| Urinary-specific agents | †Fosfomycin | 43 | 1.040 (0.844–1.283) | +4.0% | 0.711 | 1.030 (0.816–1.301) | +3.0% | 0.802 |
|  | †Nitrofurantoin | 159 | 1.025 (0.928–1.132) | +2.5% | 0.628 | 1.038 (0.936–1.152) | +3.8% | 0.476 |
| Tetracyclines | Tetracycline | 182 | 1.088 (0.991–1.195) | +8.8% | 0.077 | 1.102 (0.994–1.221) | +10.2% | 0.064 |
| *Note.* Odds ratios (OR) and annual percent change (APC = (OR − 1) × 100) are derived from binary logistic regression with year of collection as a continuous predictor. Adjusted models additionally include care setting (outpatient, hospital ward, or intensive-care unit), specimen source, patient age, and sex. APC reflects the average annual change in the odds of non-susceptibility, not the absolute annual change in resistance proportion. N tested = number of isolates with a susceptibility result for each agent. Fosfomycin and nitrofurantoin were analyzed in urinary isolates only (n = 43 and n = 159, respectively); † – urine isolates only. | | | | | | | | |
