# Supplementary material for: Laboratory-based surveillance of antimicrobial non-susceptibility in Enterobacter species over 12 years at a tertiary-care hospital in Saudi Arabia
Source: PLoS One. 2026 Oct 16;21(10):e0358963. doi: 10.1371/journal.pone.0358963 (PMC13674761; doi:10.1371/journal.pone.0358963)
Supplement: Supplementary file 7 [file pone.0358963.s008.docx]

**S7 Table.** Annual numerators, denominators, and eligibility criteria for carbapenem-resistant Enterobacterales (CRE) and difficult-to-treat resistance (DTR), 2012–2024.

| Year | CRE, n | CRE, N | CRE % | DTR, n | DTR, N | DTR % |
| --- | --- | --- | --- | --- | --- | --- |
| 2012 | 5 | 34 | 14.7 | 1 | 18 | 5.6 |
| 2013 | 17 | 124 | 13.7 | 0 | 71 | 0.0 |
| 2014 | 12 | 100 | 12.0 | 5 | 78 | 6.4 |
| 2015 | 15 | 106 | 14.2 | 5 | 102 | 4.9 |
| 2016 | 14 | 124 | 11.3 | 4 | 116 | 3.4 |
| 2017 | 21 | 110 | 19.1 | 5 | 94 | 5.3 |
| 2018 | 33 | 77 | 42.9 | 5 | 72 | 6.9 |
| 2019 | 36 | 137 | 26.3 | 11 | 117 | 9.4 |
| 2020 | 35 | 90 | 38.9 | 5 | 79 | 6.3 |
| 2021 | 65 | 144 | 45.1 | 9 | 122 | 7.4 |
| 2022 | 37 | 93 | 39.8 | 7 | 82 | 8.5 |
| 2023 | 63 | 121 | 52.1 | 23 | 114 | 20.2 |
| 2024 | 24 | 53 | 45.3 | 3 | 49 | 6.1 |
| All years | **377** | **1313** | **28.7** | **83** | **1114** | **7.5** |
| *Note.* CRE denominator (N) = isolates tested for at least one carbapenem. DTR denominator (N) = eligible isolates tested for at least one carbapenem, at least one extended-spectrum cephalosporin, piperacillin–tazobactam, and at least one fluoroquinolone. Percentages are of the corresponding annual denominator. | | | | | | |
