# Supplementary material for: Laboratory-based surveillance of antimicrobial non-susceptibility in Enterobacter species over 12 years at a tertiary-care hospital in Saudi Arabia
Source: PLoS One. 2026 Oct 16;21(10):e0358963. doi: 10.1371/journal.pone.0358963 (PMC13674761; doi:10.1371/journal.pone.0358963)
Supplement: Supplementary file 8 [file pone.0358963.s009.docx]

**S8 Table.** Timeline of antimicrobial susceptibility testing (AST) platforms, interpretive standards, and major CLSI/EUCAST Enterobacterales breakpoint revisions relevant to this study, 2012–2024.

| Year | CLSI M100 edition | EUCAST version | Major Enterobacterales breakpoint change relevant to this study |
| --- | --- | --- | --- |
| 2012 | M100-S22 | v2.0 | Study start; ESC and carbapenem breakpoints already lowered (M100-S20, 2010) |
| 2013 | M100-S23 | v3.1 | — |
| 2014 | M100-S24 | v4.0 | Cefepime: susceptible breakpoint lowered and susceptible-dose-dependent (SDD) category introduced |
| 2015 | M100-S25 | v5.0 | — |
| 2016 | M100-S26 | v6.0 | — |
| 2017 | M100 27th ed | v7.1 | — |
| 2018 | M100 28th ed | v8.1 | — |
| 2019 | M100 29th ed | v9.0 | Fluoroquinolones (ciprofloxacin, levofloxacin): breakpoints lowered; EUCAST redefined "I" |
| 2020 | M100 30th ed | v10.0 | — |
| 2021 | M100 31st ed | v11.0 | — |
| 2022 | M100 32nd ed | v12.0 | Piperacillin–tazobactam: breakpoints lowered; SDD introduced |
| 2023 | M100 33rd ed | v13.0 | Aminoglycosides (amikacin, gentamicin, tobramycin): breakpoints lowered |
| 2024 | M100 34th ed | v14.0 | Study end |
| *Note.* AST platforms: VITEK 2 Compact (primary) with selective MicroScan WalkAway confirmation; disk and gradient diffusion used selectively (see Methods). Testing panels varied over time by routine reporting practice. Breakpoint-comparable–period analyses (S9 Table) address the effect of these revisions. | | | |
