# Supplementary material for: Laboratory-based surveillance of antimicrobial non-susceptibility in Enterobacter species over 12 years at a tertiary-care hospital in Saudi Arabia
Source: PLoS One. 2026 Oct 16;21(10):e0358963. doi: 10.1371/journal.pone.0358963 (PMC13674761; doi:10.1371/journal.pone.0358963)
Supplement: Supplementary file 9 [file pone.0358963.s010.docx]

**S9 Table.** Partial-year and breakpoint-comparable–period sensitivity analyses of temporal non-susceptibility trends (annual percent change, APC).

| Agent / phenotype | Full period (2012–2024) APC | Excluding partial years (2013–2023) APC | Breakpoint-comparable period APC |
| --- | --- | --- | --- |
| Amikacin | -5.8% (p=0.009) | -4.8% (p=0.048) | 2012-2022: -7.2% (p=0.008) |
| Gentamicin | -10.0% (p=0.000) | -8.9% (p=0.000) | 2012-2022: -11.3% (p=0.000) |
| Cefepime | -9.4% (p=0.000) | -9.3% (p=0.000) | 2014-2024: -10.7% (p=0.000) |
| Piperacillin–tazobactam | +6.5% (p=0.001) | +6.5% (p=0.002) | 2012-2021: +3.0% (p=0.258) |
| Levofloxacin | +7.5% (p=0.001) | +7.9% (p=0.002) | 2012-2018: +3.6% (p=0.567) |
| Ciprofloxacin | +0.5% (p=0.765) | +1.6% (p=0.412) | 2012-2018: -19.1% (p=0.000) |
| Ertapenem | +15.7% (p=0.000) | +17.3% (p=0.000) | — |
| Imipenem | +13.0% (p=0.000) | +16.7% (p=0.000) | — |
| Meropenem | +16.6% (p=0.000) | +20.7% (p=0.000) | — |
| CRE | — | +25.1% (p=0.000) | — |
| DTR | — | +20.8% (p=0.000) | — |
| *Note.* Comparable periods restrict each agent to the interval before its major CLSI breakpoint revision (fluoroquinolones ≤2018; piperacillin–tazobactam ≤2021; aminoglycosides ≤2022) or, for cefepime, after SDD introduction (2014–2024). Carbapenem and cefepime trends and the aminoglycoside declines persisted; the levofloxacin and piperacillin–tazobactam increases attenuated to non-significance, indicating partial contribution of breakpoint revisions. | | | |
