# Supplementary material for: Laboratory-based surveillance of antimicrobial non-susceptibility in Enterobacter species over 12 years at a tertiary-care hospital in Saudi Arabia
Source: PLoS One. 2026 Oct 16;21(10):e0358963. doi: 10.1371/journal.pone.0358963 (PMC13674761; doi:10.1371/journal.pone.0358963)
Supplement: Supplementary file 10 [file pone.0358963.s011.docx]

**S10 Table.** Patient-level cluster-robust and first-isolate-per-patient sensitivity analyses (1,319 isolates from 1,000 patients).

| Agent / phenotype | Unadjusted APC (p) | Cluster-robust SE (p) | First isolate per patient APC (p) |
| --- | --- | --- | --- |
| Amikacin | -5.8% (p=0.009) | p=0.029 | -6.4% (p=0.014) |
| Gentamicin | -10.0% (p=0.000) | p=0.000 | -10.4% (p=0.000) |
| Cefepime | -9.4% (p=0.000) | p=0.000 | -8.8% (p=0.000) |
| Piperacillin–tazobactam | +6.5% (p=0.001) | p=0.006 | +6.6% (p=0.003) |
| Levofloxacin | +7.5% (p=0.001) | p=0.013 | +5.4% (p=0.047) |
| Ciprofloxacin | +0.5% (p=0.765) | p=0.843 | -0.2% (p=0.931) |
| Ertapenem | +15.7% (p=0.000) | p=0.000 | +12.3% (p=0.000) |
| Imipenem | +13.0% (p=0.000) | p=0.000 | +13.6% (p=0.000) |
| Meropenem | +16.6% (p=0.000) | p=0.000 | +15.2% (p=0.000) |
| CRE | +22.8% (p<0.001) | p=0.000 | +22.4% (p=0.000) |
| DTR | +16.4% (p<0.001) | p=0.001 | +10.1% (p=0.029) |
| *Note.* Cluster-robust standard errors were clustered on the patient identifier; the first-isolate analysis retained one (earliest) isolate per patient (n = 1,000). Trend directions and significance were preserved throughout. | | | |
