# Supplementary material for: Laboratory-based surveillance of antimicrobial non-susceptibility in Enterobacter species over 12 years at a tertiary-care hospital in Saudi Arabia
Source: PLoS One. 2026 Oct 16;21(10):e0358963. doi: 10.1371/journal.pone.0358963 (PMC13674761; doi:10.1371/journal.pone.0358963)
Supplement: Supplementary file 11 [file pone.0358963.s012.docx]

**S11 Table.** Species-level distribution within the other Enterobacter species group (n = 108).

| Reported identification | n | % of other Enterobacter |
| --- | --- | --- |
| *Enterobacter species (not further differentiated)* | 41 | 38.0% |
| *Enterobacter agglomerans group* | 26 | 24.1% |
| *Enterobacter gergoviae* | 13 | 12.0% |
| *Enterobacter sakazakii* | 10 | 9.3% |
| *Enterobacter amnigenus (biogroup 1)* | 6 | 5.6% |
| *Enterobacter cancerogenus* | 6 | 5.6% |
| *Enterobacter intermedius* | 5 | 4.6% |
| *Enterobacter amnigenus (biogroup 2)* | 1 | 0.9% |
| Total | **108** | **100.0%** |
| *Note.* Identifications as reported by the automated systems (VITEK 2 with selective MicroScan). Several taxa have since been reclassified (e.g., Pantoea, Pluralibacter, Cronobacter, Kosakonia, Lelliottia). | | |
